# Supplementary material for: Feasibility of Find cases Actively, Separate safely and Treat effectively (FAST) strategy for early diagnosis of TB in Nepal: An implementation research
Source: PLoS One. 2021 Oct 26;16(10):e0258883. doi: 10.1371/journal.pone.0258883 (PMC8547636; doi:10.1371/journal.pone.0258883)
Supplement: Attachment — Submitted filename: review plos one.docx [file pone.0258883.s003.docx]

**Introduction**

Line no 25: Please remove visiting

Line no 25: It should be Tb infections..

Line no 28: “This study also explored barriers and enablers that

influence the proper implementation of the FAST strategy in Nepal”.. it sounds much better if we rephrase it as “In addition, this study also…”

line no 36: change was to were.. change to as per the requirement

line 44: please rephrase the sentences “freely available of services”

line no 55: does “ill” mean people were sick from TB or were they just infected with TB bacilli? Not all TB infected people become sick from TB.

line no 56: “In 2017, 1.6 million people were died” should be rewritten was 1.6 million people died from TB.

line no 58-60: please rephrase the sentences.

Line no 64-65: “As per the estimation made by WHO, Nepal is still missing around 12,000 to 13,000 incidences”—is this the detection gap? Incidences or cases?

Line 66-69: please rephrase the sentences as its confusing.

Line no 71: spread in health facility or community?

Line no 106: change to different settings

Line no 130-131: PI collected data using FGD guideline and KII tool.

Line no 139: delete be.

Line no 151: A total of fourteen health care providers,: was it 14 health care providers or total participants were 14?.. rephrase as there were total 14 participants, which included..

Line 163: “high OPD patient flow hospitals.” Please rephrase this sentences

**Result section**

The result section needs to be rewritten. Its quite difficult to comprehend some sentences.

**General comments**

Proof reading has to be done to check for grammar and sentencing.
