# Supplementary material for: Feasibility of Find cases Actively, Separate safely and Treat effectively (FAST) strategy for early diagnosis of TB in Nepal: An implementation research
Source: PLoS One. 2021 Oct 26;16(10):e0258883. doi: 10.1371/journal.pone.0258883 (PMC8547636; doi:10.1371/journal.pone.0258883)
Supplement: Attachment — Submitted filename: Response to Reviewers.docx [file pone.0258883.s004.docx]

**Author’s response to reviews**

**Title:** **Feasibility of Find cases Actively, Separate safely and Treat effectively (FAST) strategy for early diagnosis of TB in Nepal: An implementation research**

**Authors:**

Sagun Paudel ([mail4sagun@gmail.com](mailto:mail4sagun@gmail.com))

Retna Siwi Padmawati ([rspadmawati@ugm.ac.id](mailto:rspadmawati@ugm.ac.id))

Ashmita Ghimire ([ashmi.gr8@gmail.com](mailto:ashmi.gr8@gmail.com))

Choden Lama Yonzon ([yonzon.chhoden.cy@gmail.com](mailto:yonzon.chhoden.cy@gmail.com))

Yodi Mahendradhata ([yodi_mahendradhata@ugm.ac.id](mailto:yodi_mahendradhata@ugm.ac.id))

Shyam Sundar Budhathoki, MBBS, MD, MPH

Academic Editor

PLOS ONE

**Subject: Re-submission of the revision of manuscript PONE-D-20-20684**

Respected Sir,

Thank you for the opportunity to revise our manuscript entitled “Feasibility of Find cases Actively, Separate safely and Treat effectively (FAST) Strategy for Early Diagnosis of TB in Nepal: An Implementation Research” PONE-D-20-20684. We are thankful to both reviewer 1 and reviewer 2 for the constructive comments and feedback on our manuscript.

We feel that the suggestions received from reviewers are insightful and helped us in improving the manuscript. We have made attempt to fully address these comments and incorporate the feedback in the revised manuscript and believe our revised manuscript represents a significant improvement over our initial submission. Please find attached a response to reviewers for your kind consideration. We hope that you find our responses satisfactory.

In addition, I would like to kindly inform you that the available data sets for this manuscript are also attached in supporting files.

Many thanks again.

Sincerely,

Sagun Paudel, MPH

**Response to Reviewers**

**Reviewer 1**

**Point 1**: Title – this needs revision as it does not encompass the full definition of FAST (see above first line of comments) as FAST is not merely about early diagnosis, but early diagnosis coupled with effective treatment.

**Response 1:** Agreed with comment. Updated as per suggestion.

**Point 2:** Abstract Needs full definition of FAST as above

**Response 2:** This has been revised in Manuscript.

**Point 3:** Sentence beginning line 23 needs to incorporate importance of effective treatment and would not focus on OPD here as FAST was initially designed for inpatient settings

**Response:** 3: The sentence now reads: Feasibility of ……. among inpatient settings as well as OPD patients and prevent TB infections in hospital settings.

**Point 4:** Introduction last three sentences read in a disjointed fashion – suggest revising
**Response 4:** We have revised the sentence and has updated in manuscript.

**Point 5**: Methods (would remove materials as not relevant to this study type) – state prospective vs retrospective study

**Response 5:** ‘Materials’ removed, and retrospective added.

**Point 6:** Focus rather than focused group discussion

**Response 6:** Corrected.

**Point 7:** Methods section is too brief – suggest shortening introduction to enable addition of text re: healthcare setting/country context and analytic methodology

**Response 7**: Study setting/healthcare setting and data analysis methodology has been added.

**Point 8:** Results are not well organized and primarily consist of lists, suggest revising
**Response 8:** This has been revised as per the suggestions.

**Point 9:** Conclusion – sentence about hospital ownership is important but requires a bit more explanation e.g. hospital ownership (as opposed to?)

**Response 9: Conclusion is revised as , ‘‘……**Hospital ownership is essential to mobilize health workers, improve client flow system and proper space management for FAST services.’’

**Point 10:** Update data in first sentences based on WHO 2020 report

**Response:** Updated with latest report.

**Point 11:** Line 56- unclear relevance of latent TB for this study- suggest removing

**Response 11:** Removed as suggested

**Point 12:** Line 57 – 44% of cases – I think this refers to active rather than latent TB? Would move or remove since you later discuss Nepal specific data.

**Response 12:** Removed as suggested

**Point 13:** Line 65 – would rephrase incidences as people who become sick with TB

**Response 13:** Rephrased as ‘‘people who become sick with TB’’ as suggested.

**Point 14:** Lines 66-68 – unclear why you are discussing the 2013-2015 data since this study was done subsequently

**Response 14:** Deleted as the 2013-2015 data is irrelevant

**Point 15:** Line 68-69 – this statistic repeats the earlier statistic re: 12-13 000 cases missed/year

**Response 15:** The duplicated statistic has been removed and rephrased.

**Point 16:** Lines 70-71 – suggest rephrasing

**Response 16:** Rephrased as ‘‘Early case detection at the health facility is important to reduce the suffering time of patient and to reduce the spread of infection at health facility level.’’
**Point 17:** Line 72 – as mentioned above please define FAST as per Barrera, Nardell IJTLD reference and cite this

**Response 17:** Definition of FAST revised as Find cases Actively, Separate safely and Treat effectively strategy and cited clearly

**Point 18:** Line 74 – early diagnosis to enable prompt initiation of effective treatment

**Response 18:** Revised as suggested
 **Point 19:** Line 75-76 - would replace chest symptomatic with potentially infectious patient

**Response 19:** Replaced as suggested.

**Point 20:** Line 77-79 – would remove as FAST has not been proven to be the best strategy as stated

**Response 20:** Removed as suggested.

**Point 21:** Line 79-81 - would state that FAST was initially designed for implementation in inpatient settings because of the central importance of early effective treatment playing a major role in decreasing transmission

**Response 21:** Corrected as above.

**Point 22:** Line 84-85 – would remove by healthcare workers and cough officers as optimal approach for implementing FAST has not yet been established

**Response 22:** The sentence ‘‘by health care providers and cough surveillance officers.’’ removed.

**Point 23:** Lines 92-94 – this describes the study results and so should not be in the introduction
**Response 23:** Removed.

Methods
**Point 24:** Line 98 – revise sentence re: study design

**Response 24**: statement revised as ‘‘This study was an implementation research that used qualitative study design.’’

**Point 25:** Line 99 – focus rather than focused group

**Response 25**: corrected as focus group discussion

**Point 26:** Lines 100-101 – please briefly describe purposive sampling method/describe how participants were recruited

**Response 26:** Brief information added ‘‘Respondents were purposively selected from hospitals who were directly involved in the implementation of FAST activities and currently working on FAST activities.’’

**Point 27 :** Lines 106-107 – difference between 1) and 2) is not entirely clear- please clarify.

**Response 27:** Difference between 1) & 2) added as ‘‘1) hospitals in difference setting where FAST strategy was implemented, 2) Province and district level health authorities where planning, management, supply related activities are managed’’

**Point 28 :** Lines 108-110 – this answers some of the prior question re: purposive sampling, would move

**Response 28:** Rephrased as …..because these hospitals can represent all the FAST implementing hospitals as they shared the same goal for implementation of FAST approach in Nepal.’’

**Point 29 :** Line 114 - you say similarly but test appears to be more in keeping with ‘In contrast..’ and you mention this is a community rather than referral hospital so unclear what is similar?

**Response 29:** Sentence rephrased as ….’’Another study ares…………’’

**Point 30:** Line 119-120 – describe how these stakeholders were selected/sampled and from which healthcare contexts?

**Response 30:** . Information updated as ‘‘The purposively selected TB focal person, district and province TB coordinators were interviewed who were primarily responsible to monitor, support and supervise the FAST activities at district and province level who were directly involved in planning, supervision and monitoring of the FAST implementing hospitals…’’

**Point 31:** Line 124 – what qualitative research instruments? Explain this? Also you have not defined KII earlier.

**Response 31:** Information added as…’The face-to-face interview with key informants was conducted. The instruments consist of a semi-structured questionnaire and FGD guideline. The Consolidated Framework for Implementation Research (CFIR) domains; inner setting, individual involved, and implementation process were used to prepare the interview tools…’

**Point 32:** Line 125 – KII not defined before

**Response 32:** KII is defined.

**Point 33:** Line 128 – clear that patient interviews were exit interviews- how and when were interviews with health workers performed?

**Response 33:** Information updated as ‘‘….interview was taken with healthcare workers after completion of their regular screening process of patients at hospital. Similarly, client exit interview was taken among patients to explore the client’s perspective regarding the FAST approach. Those patients who were screened for TB at hospital by using FAST approach were interviewed after completion of their screening process at hospital….’’

**Point 34:** Line 129 – how were these instruments developed?

**Response 34:** ‘‘The Consolidated Framework for Implementation Research (CFIR) domains; inner setting, individual involved, and implementation process were used to prepare the interview tools.’’

**Point 35:** Lines 133- 137 – requires more detail e.g. inductive versus deductive analysis and reference for thematic analysis e.g. Braun and Clarke
Context of how FAST implementation occurred in the country and at your sites should be described in the methods, including how was responsible for implementing FAST
Also need description of what FAST implementation means e.g. screening all patients with cough vs other respiratory symptoms? Did all get Xpert? Did some get chest x-ray?
**Response 35:** The context of how FAST implementation is mentioned in study setting

**Results**
**Point 36:** Characteristics – should report numbers for all types of data including focus groups. For KIIs, would not list districts but FAST implementing sites as you do for the patients
I recommend a paragraph describing how and which themes were derived before describing findings, currently unclear if these subheadings are the themes based on inductive versus deductive analysis

**Response 36:** Paragraph added before describing results and the information on analysis is also added to data analysis part.

**Point 37:** Line 164 – what does good result re: case notification mean?

**Response 37:** Actually, it means FAST helps to increase case notification of TB at hospitals. This is now paraphrased.

**Point 38:** Line 165 – what does implemented as a non-costed intervention mean?

**Response 38:** Actually, it means FAST helps to increase case notification of TB at hospitals. This is now paraphrased.

Point 39: Lines 168-179 – see above comment re: context of how FAST implementation occurred in the country and at your sites should be described in the methods

**Response 39**: Briefly added the context.

**Point 40 :** Line 180 – when you say respondent, should specify from which participant group

**Response 40**: Added as suggested.

**Point 41:** Line 186 – crowded not crowed

**Response 41:** Corrected.

**Point 42:** Line 197-199 – clarify you mean this could be changed with administrative buy-in/support

Response 42: Yes, it is. Changes in the administrative process as well as structural (physical) arrangement of hospitals can enable the FAST implementation process.

Point 43: Lines 217-218 – please clarify what this means: case identification was not as expected and low number of patients were screened for TB

Response 43: The policy makers expected that FAST will increase the significant numbers of cases promptly, but practically it was not happened initially. After mobilizing dedicated staff and space for FAST screening, the case notification at hospital was increased.

Point 44: Line 223 – remove stigmatizing term suspected, also in line 272, 284

Response 44: Removed as per suggestion.

**Point 45:** Line 232 – what about initiation of treatment?

**Response 45:** One sentence added based on the finding. The treatment initiation process was quickly initiated when cases were diagnosed via FAST approach at hospital.

**Point 46:** Line 249 – which hospital does this quote refer to?

**Response 46:** This is related to Government hospitals

**Point 47:** Lines 289-300 – is this all hospitals/some hospitals? Which respondents?

**Response 47:** This issues was specially highlighted by health workers who are working in government hospital.

**Point 48:** Line 299 – who were these patient navigators?

**Response 48:** The patient navigators are the support staffs mobilized at hospital to provide information to patients and guide them to move from one room to another room to get timely services. This information is also added to the manuscript.

**Point 49:** Line 385 – again explain non-costed intervention? Integrated into routine care?

**Response 49:** FAST approach is new concept which was initiated in only few hospitals without mobilizing additional financial resources so they (program managers) called it as a non-costed intervention and It was implemented within the routine healthcare delivery service, TB screening and treatment protocol.

**Point 50:** Line 400 – what are IEC materials?

**Response 50:** IEC materials are booklets, wall charts, pamphlets and information sheets on FAST approach.

**Discussion
Point 51:** Line 437-439 – unclear relevance of this citation?

**Response 51:** Removed in manuscript.

**Point 52 :** Line 461-463 – similar to earlier, unclear relevance of this citation?

**Response 52:** unclear relevance of this citation is removed from manuscript.

**Point 53:** Line 471-473 – this contradicts what you say re: need for space and dedicated staff?

**Response 53:** It was found that the FAST approach can be basically implemented by mobilization of the existing staffs and managing the existing space for services (screening, separating, ventilation). So, to initiate the FAST extra financial cost is not required.

**Point 54:** Line 485 – repetition with earlier section at line 455 re: Xpert

**Response 54:** Repeated result is removed from manuscript.

**Point 55:** Lines 489-490 – this citation does not refer to FAST so clarification needs to be made

**Response 55:** The study conducted in Pakistan shows that the systematic screening of TB symptomatic patients helps to identify the undiagnosed cases there, as FAST is also a systematic approach of infection prevention and case identification it seems relevant to the screening process.

**Point 56:** Line 493 – rephrase, I think you mean prevent transmission?

**Response 56:** This has been revised.

**Point 57:** Lines 506-517 – this is the first mention of CFIR (should cite Damschroeder 2009) and this discussion is inadequate. If CFIR was used to guide data collection this should be in the methods and the themes described should discuss CFIR domains e.g. inner/outer setting more clearly

**Response 57:** The Consolidated Framework for Implementation Research (CFIR) domains; inner setting, individual involved, and implementation process were used to prepare the interview tools. This is now added to the data collection and analysis section of manuscript.


**Conclusion**
**Point 58:** Lines 522-523 - need to rephrase

**Response 58:** rephrased as ‘‘…..The findings of this research conclude that the implementation set-up and service delivery mechanism at hospitals is not yet well practiced as a routine process for the FAST approach however it can be managed if hospital administration takes responsibility to institutionalization this approach….’’

**Point 59:** Lines 533-534 – you do not provide information that FAST reduces transmission or even provide data about number of cases diagnosed so cannot conclude this.

**Response 59:** Agreed and updated in manuscript.

**Reviewer #2**

Introduction
**Point 1:** Line no 25: Please remove visiting

**Response 1**: Removed.

**Point 2:** Line no 25: It should be Tb infections..

**Response 2:** Corrected.

**Point 3:** Line no 28: “This study also explored barriers and enablers that
influence the proper implementation of the FAST strategy in Nepal”.. it sounds much better if we rephrase it as “In addition, this study also…”

**Response 3**: Corrected.

**Point 4:** line no 36: change was to were.. change to as per the requirement

**Response 4:** We have revised the sentence and has updated in manuscript.

**Point 5:** line 44: please rephrase the sentences “freely available of services”

**Response 5:** we have revised the sentence and has updated in manuscript.

**Point 6 :** line no 55: does “ill” mean people were sick from TB or were they just infected with TB bacilli? Not all TB infected people become sick from TB.

**Response 6**: Corrected.

**Point 7:** line no 56: “In 2017, 1.6 million people were died” should be rewritten was 1.6 million people died from TB.

**Response 7** : We have revised the sentence.

**Point 8:** line no 58-60: please rephrase the sentences.

**Response 8:** Rephrased.

**Point 9:** Line no 64-65: “As per the estimation made by WHO, Nepal is still missing around 12,000 to 13,000 incidences”—is this the detection gap? Incidences or cases?

**Response 9:** True, this is the detection gaps in the total estimated cases.

**Point 10:** Line 66-69: please rephrase the sentences as its confusing.

**Response 10:** Rephrased.

**Point 11:** Line no 71: spread in health facility or community?

**Response 11:** Both, at health facility and ultimately at community level.

**Point 12:** Line no 106: change to different settings
**Response 12:** Changed.

**Point 13:** Line no 130-131: PI collected data using FGD guideline and KII tool.
**Response 13:** Corrected.

**Point 14:** Line no 139: delete be.
**Response 14:** Deleted

**Point 15:** Line no 151: A total of fourteen health care providers,: was it 14 health care providers or total participants were 14?.. rephrase as there were total 14 participants, which included.
**Response 15:** This sentence is corrected as ‘‘There were total 27 participants, which included health care providers, hospital managers, district focal persons, provincial team leader, program coordinators and patients.’’
 **Point 16:** Line 163: “high OPD patient flow hospitals.” Please rephrase this sentence
**Response 16:** Rephrased.
 **Result section**
**Point 17:** The result section needs to be rewritten. It’s quite difficult to comprehend some sentences.

**Response 17:** Result is rephrased.
