# Supplementary material for: Feasibility of Find cases Actively, Separate safely and Treat effectively (FAST) strategy for early diagnosis of TB in Nepal: An implementation research
Source: PLoS One. 2021 Oct 26;16(10):e0258883. doi: 10.1371/journal.pone.0258883 (PMC8547636; doi:10.1371/journal.pone.0258883)
Supplement: Attachment — Submitted filename: Response to Reviewers.docx [file pone.0258883.s005.docx]

**Author’s response to reviews**

**Title:** **Feasibility of Find cases Actively, Separate safely and Treat effectively (FAST) strategy for early diagnosis of TB in Nepal: An implementation research**

**Authors:**

Sagun Paudel ([mail4sagun@gmail.com](mailto:mail4sagun@gmail.com))

Retna Siwi Padmawati ([rspadmawati@ugm.ac.id](mailto:rspadmawati@ugm.ac.id))

Ashmita Ghimire ([ashmi.gr8@gmail.com](mailto:ashmi.gr8@gmail.com))

Choden Lama Yonzon ([yonzon.chhoden.cy@gmail.com](mailto:yonzon.chhoden.cy@gmail.com))

Yodi Mahendradhata ([yodi_mahendradhata@ugm.ac.id](mailto:yodi_mahendradhata@ugm.ac.id))

To, Natasha McDonald, PhD

Associate Editor

PLOS ONE

**Subject: Re-submission of the revision of manuscript PONE-D-20-20684R1**

Respected Sir,

Thank you for your consideration of our manuscript entitled “Feasibility of Find cases Actively, Separate safely and Treat effectively (FAST) Strategy for Early Diagnosis of TB in Nepal: An Implementation Research” PONE-D-20-20684R1. We have reviewed the comments of the reviewers and have thoroughly revised our manuscript We are thankful to both reviewer 3 and reviewer 4 for the constructive comments and feedback on our manuscript.

We found the comments, correction and suggestions received from reviewers are insightful and helped us in improving the manuscript. We have made attempt to fully address these comments and incorporate the feedback in the revised manuscript and believe our revised manuscript represents a significantly improved. Please find attached a point-by-point response to reviewers for your kind consideration. We hope that you find our responses satisfactory, and that the manuscript will be accepted for publication.

Many thanks again.

Sincerely,

Sagun Paudel, MPH

**Response to Reviewers**

**Reviewer #4**

**Abstract**

**Point 1:** line 24 OPD – change to full wording

**Response:** This has been updated with ‘Out-patient department’

**Point 2:** line 35 – rephrase “study identified that the current setting of implementation…”

**Response:** Updated as per suggestion.

**Point 3:** line 37-38 – rephrase – “…showed that hospital ownership is crucial/is important for mobilizing staff (not staffs)….”

**Response:** Rephrased as per suggestion.

**Point 4:** line 38 – remove “that”

**Response:** Removed.

**Point 5:** line 40 - cartridge not cartilage

**Response:** Corrected.

**Point 6:** line 40 – manpower (not fair with women) = human resources

**Response:** Added as per suggestion.

**Point 7:** line 44 – rephrase “… effective function of the strategy”

**Response:** Rephrased as per suggestion.

**Introduction**

**Point 8:** line 50 – rephrase, it is not top ten it is among top ten, caused by single infectious agent, update WHO report date – 2017 to the newer version.

**Response:** Updated as per latest report.

**Point 9:** line 52 - remove “which is steady in 2018”

**Response:** Removed.

**Point 10:** line 57 – add “… among the leading causes of death in the country”

**Response:** Added as per suggestion.

**Point 11:** line 64 - rephrase – “Early case detection is important to interrupt infection transmission chain within healthcare facilities and community, in addition to reduce time of patients` suffering.”

**Response:** Rephrased as per suggestion.

**Point 12**: line 63 – NTC needs full wording

**Response:** Updated National Tuberculosis Control Center

**Point 13**: line 65 – change activity to process.

**Response:** Changed as per suggestion.

**Point 14**: line 68-70 - remove sentence starting with “Fast was initially…”

**Response:** Removed as per recommendation

**Point 15**: line 71 – figure 1 – figure misses “No” in the upper right option of current cough and the options of two conditions such as: 1. Signs and symptoms – yes / chest x-ray –normal; 2. Signs and symptoms –no / chest x-ray abnormal; - what happens in such situations?

**Response:** Figure 1 has been updates and modified to make it simple.

**Point 16**: line 73 – define DR- TB

**Response:** Added as Drug-resistant tuberculosis (DR-TB)

**Point 17**: line 77 – change early diagnosis to early detection

**Response:** Updated as per suggestion.

**Point 18**: line 82-88– rephrase to – “In order to suggest National Tuberculosis control program the areas which are crucial to be improved to strengthen the health facility-based diagnosis and prompt treatment of TB, we aimed to explore organizational, technical and financial factors needed to implement FAST activities in four hospitals.”

**Response:** Updated as per recommendation.

**Materials and methods**

**Point 19**: line 97 – define criteria for “purposively selected patients” – meaning they were selected based on…?

**Response:** Those patients, who attended the out-patient department with chest symptomatic features and screened for TB were selected for the interview purpose.

**Point 20**: line 92 – include FGD abbreviation with focus group discussion

Response: Updated as focus group discussion.

**Point 21**: Study setting – start paragraph with line 110 – “The FAST is a global fund-supported activity, where various non-governmental organizations are providing technical support to the …..” continue with line 106, “The FAST strategy was prepared by ….”

**Response**: Changed as per the suggestion.

**Point 22**: line 115 change “The FAST approach” to “As FAST approach”

**Response**: Added as per suggestion.

**Point 23**: line 117 change “separated presumptive TB cases to collect sputum for GeneXpert and diagnosed patients were enrolled for the treatment”

**Response:** Corrected.

**Point 24**: line 120 – difference or different?

**Response:** It is different, Corrected.

**Point 25**: line 125 – remove part of the sentence starting from “because these hospitals…”

**Response**: Removed as per suggestion.

**Results**

**Point 26:** line 188 remove “purposively”

**Response:** Removed.

**Point 27:** line 190 – define groups clearly, eg. i) implementation context, ii) identified factors for feasibility iii) barriers and enablers iv) way forward for the program implementation

**Response:** Result groups are defined as per suggestion.

**Point 28:** Table 1 – Patient who is 40 years old, is over 40 or below 40? Define age categories like ≥40 and <40 at least. Or 18-39 / 40 and up… Same goes to work experience length.

**Response:** Corrected as ≥ 40 and ≥ 17 in age and work experiences respectively.

**Point 29**: line 197 – respondent or respondents?

**Response:** It was respondents, corrected.

**Point 30**: line 199-200 rephrase

**Response:** Rephrased as **‘‘**National stakeholders described that FAST is implemented as a non-costed intervention as there is no financial implication for program implementation, where TB screening and treatment services are offered at different hospitals.’’

**Point 31**: line 219 – change “So, it is difficult” to “Therefore it is difficult to pick the patient, compared to the Teaching hospital and Achham hospital.”

**Response:** Revised as per suggestion.

**Point 32:** line 222-225 rephrase whole paragraph “…and physical space can be managed inside the hospitals.”

**Response:** Rephrased as **‘‘**Participants explained that the implementation setting was not readily structured as per the requirements however they strongly argued that the hospital setting, and service operation process can be easily modified as per FAST guideline to implement the FAST approach and physical space can manage inside hospitals.’’

**Point 33:** line 228 –include “[sic]” at the end of every quote, indicating there might be grammatical problems and you are not interfering with corrections.

**Response:** Corrected.

**Point 34:** line 231 – change “… increased in implementing” to “… increased in the selected hospitals.”

**Response:** Revised as per suggestion.

**Point 35:** line 232 remove chest and change “… are prioritized to be examined faster.”

**Response:** Changed accordingly.

**Point 36**: line 242 – “as per the expectation at the initial stage…”

**Response:** Corrected.

**Point 37:** line 243 – “…results show that the TB…”

**Response:** Corrected per suggestion**.**

**Point 38**: line 246 – rephrase – “…at the hospitals was performed per protocol, however…”

**Response:** Updated as per suggestion.

**Point 39**: paragraph lines 250-259 – shorten too many repetitions. Needs just a few sentences.

**Response:** Rephrased as **‘‘**As the FAST approach consists of series of activities in the hospital setting, our study identified that the actively screening, counseling, and sputum examination was conducted as per the framework of FAST strategy however separation of the patient from the queue and place them in the isolated or well-ventilated room was not followed as per the requirement of the program. It was found that the diagnosed cases were properly enrolled for DOTS. As per the FAST framework, the main problem was identified on the separation of patients. This study identified that the treatment of diagnosed TB cases was initiated timely and the patients were enrolled at DOTS therapy within hospitals.’’

**Point 40**: lines 272- 275 – unclear, please rephrase, avoid repetition of the specific words.

**Response:** Rephrased as **‘‘**Participants repeatedly stressed that strong hospital ownership is a fundamental factor for staff mobilization, arrangement of the client flow system, registration desk, and waiting room. Similarly, hospital ownership is required for overall program implementation, monitoring, recording, reporting, and evaluation of the program. We identified that the hospital ownership was established in a community hospital and NGO-operated hospital to mobilize local resources for FAST whereas the government hospital didn’t realize it.’’

**Point 41:** line 282 – change stressed to defined

**Response:** Corrected.

**Point 42**: line 286 – rephrase “… limited space overcrowded with patients…”

**Response:** Corrected as per suggestion.

**Point 43**: line 311 – Respondent or respondents?

**Response:** Corrected as respondents.

**Point 44**: line 324 – explain the meaning of “birami sahayogi”.

**Response:** Birami sahayogi is the administrative staff mobilized to support patients for searching service rooms inside hospitals.

**Point 45**: line 327 – rephrase “… support staffs mobilized..” to “… support personnel mobilized…”

**Response:** Corrected as per suggestion.

**Point 46:** line 348 – remove “... so it was not necessary for FAST”, but would be better to rephrase the whole sentence.

**Response:** Removed as per suggestion and rephrased.

**Point 47**: line 361- result waiting time is turn-around time or reporting time. Change “until the next day.”

**Response:** Changed.

**Point 48**: lines 362-363 – rephrase to “… the patients within a day, however…”

**Response:** Corrected.

**Point 49**: line 375 – add “the” – “… to the other institutions...”

**Response:** Corrected.

**Point 50**: line 386 – rephrase – “The results showed absence of uniformity on counseling….”

**Response:** Corrected.

**Point 51**: line 408 – rephrase voiced to pointed out. Remove “is required” at the end of the sentence.

**Response:** Corrected.

**Point 52:** line 425 – manpower = human resources / work force

**Response: Corrected.**

**Point 53:** line 428 – full wording for IEC.

**Response:** Added. Information, education, and communication (IEC)

**Point 54:** line 438 - “… DOTS service …. was also noticed as the enabling factor”

**Response:** Corrected. DOTS service in the same hospital were also noticed as an enabling factor.

**Point 55:** line 441 - change stressed to highlighted

**Response:** Corrected.

**Point 56:** line 453 – maybe easy adoptable instead of “doable and possible”

**Response:**

**Discussion** – Overall, this section is too big. There are many unnecessary parts, please shorten considerably. Do not repeat results as they already are in the section above.

**Response**: Attempted to reduce the duplicate content from results.

**Point 57:** line 464 “… support are fundamental factors…”

**Response:** Corrected.

**Point 58:** line 466 – “… prevent infection transmission in a hospital setting…”

**Response:** Corrected as per suggestion.

**Point 59:** line 472 – “… is necessary for the FAST strategy functioning, so the hospitals will be obliged to implement the process in healthcare facilities.”

**Response:** This has been revised.

**Point 60:** line 485 – “… delay in the maintenance..,”

**Response:** Corrected.

**Point 61**: line 502 – “…barrier to the proper…”

**Response:** Corrected.

**Point 62:** line 507 – “… and timely maintenance…”

**Response:** Revised.

**Point 63:** line 508 – “… were considered as barriers for…”

**Response:** Revised.

**Point 63**: lines 517-520 – remove and start “We could not access…”

**Response:** Removed and corrected.

**Point 64:** lines 529 -533 – remove and start “Our study showed that CFIR….”

**Response:** Removed as per suggestion.

**Conclusion**

**Point 65:** line 542 – rephrase “This study provides valuable information for implementation and feasibility of FAST strategy in Nepal”.

**Response:** Added as per suggestion.

**Reviewer #3:**

**Point 1:** It will be helpful to describe better the variables considered under organizational, technical & financial factors in the data collection and analysis section-line 123

**Response**: Variables considered for this study has been updated. Please refer 180 to 185.

**Point 2:** Line 151-157 should all be part of the methodology

Response: It was updated as per the suggestion of previous reviewer.

**Point 3:** The conclusion: line 529-532 especially linking the conclusion with early diagnosis is not supported by the study.

**Response:** Revised.
