# Supplementary material for: Feasibility of Find cases Actively, Separate safely and Treat effectively (FAST) strategy for early diagnosis of TB in Nepal: An implementation research
Source: PLoS One. 2021 Oct 26;16(10):e0258883. doi: 10.1371/journal.pone.0258883 (PMC8547636; doi:10.1371/journal.pone.0258883)
Supplement: Attachment — Submitted filename: Response to Reviewers.docx [file pone.0258883.s006.docx]

**Author’s response to reviews**

**Title:** **Feasibility of Find cases Actively, Separate safely and Treat effectively (FAST) strategy for early diagnosis of TB in Nepal: An implementation research**

**Authors:**

Sagun Paudel ([mail4sagun@gmail.com](mailto:mail4sagun@gmail.com))

Retna Siwi Padmawati ([rspadmawati@ugm.ac.id](mailto:rspadmawati@ugm.ac.id))

Ashmita Ghimire ([ashmi.gr8@gmail.com](mailto:ashmi.gr8@gmail.com))

Choden Lama Yonzon ([yonzon.chhoden.cy@gmail.com](mailto:yonzon.chhoden.cy@gmail.com))

Yodi Mahendradhata ([yodi_mahendradhata@ugm.ac.id](mailto:yodi_mahendradhata@ugm.ac.id))

To, Frederick Quinn
Academic Editor
PLOS ONE

**Subject: Re-submission of the revision of manuscript PONE-D-20-20684R2**

Respected Sir/Madam,

Thank you very much for this opportunity to submit a revised draft of manuscript entitled “Feasibility of Find cases Actively, Separate safely and Treat effectively (FAST) Strategy for Early Diagnosis of TB in Nepal: An Implementation Research” PONE-D-20-20684R2. We really appreciate the time and effort provided by Editors and reviewers and providing insightful feedback to improve our paper. We have reviewed the corrections and feedback received and and have thoroughly revised our manuscript.

We found the comments, correction and suggestions received from reviewers are insightful and helped us in improving the manuscript. Please find attached a point-by-point response to reviewers for your kind consideration. We hope that you find our responses satisfactory, and that the manuscript will be accepted for publication.

Many thanks again.

Sincerely,

Sagun Paudel, MPH

**Response to Reviewers**

**Reviewer #4:**

**Line 25 –** remove “settings” - change to “… TB transmission in hospitals”

**Response:** This has been updated.

**Line 36 –** “…current settings…”

**Response:** Changed.

**Line 60-61 –** re-phrase: “Tuberculosis is a major public health problem in Nepal, as it is ranked as the sixth among the leading causes of death in the country.”

**Response:** Rephrased as per suggestion

**Line 67 –** add to deletion - “—tome of patient” (there is a repetition)

**Response:** Deleted the repeated words ‘time of patient’

**Line 93 –** insert full stop after “...improve.”

**Response**: Added.

**Line 126-** delete “…to the...” (There is a repetition)

**Response:** Removed

**Line 150 –** correct – “Another study area/site…”

**Response:** Corrected.

**Line 243 –** Remove – “So it is difficult” and start with “Therefore it is difficult”

**Response:** Removed.

**Line 248 –** Add comma after “requirements” change full stop to comma after “setting”

**Response:** Changed.

**Line 253 –** Correct “…physical space can be managed inside the hospitals”

**Response**: Corrected.

**Line 261-** re-correct – “…as well as symptomatic patients are prioritized to examine faster.” Remove repetition.

**Response:** Removed.

**Line 282 –** Divide the sentence, use full stop after “strategy.” And start following sentence with “However separation…”

**Response:** Corrected

**Line 409** – change waiting time to reporting time

**Response:** Changed.

**Line 536** – change waiting time to reporting or turn-around time

**Response:** Changed to turn-around time.

**Line 562** – remove “a” “were considered as barriers”

**Response:** Removed

Lines 228, 237, 246, 256, 265, 268, 323, 339, 347, 380, 440, 453, 500 – at the end of each quote include [sic], e.g. “‘‘It is a very basic matter,…. Space management, a supportive laboratory staff, GeneXpert and one dedicated staff is enough to implement FAST. [sic] ’’ (Hospital focal person, community hospital, KII)

**Response:** Added
